# Kampo Medicine Treatment for Advanced Pancreatic Cancer: A Case Series

**DOI:** 10.3389/fnut.2021.702812

**Published:** 2021-08-12

**Authors:** Masayuki Shimizu, Shin Takayama, Akiko Kikuchi, Ryutaro Arita, Rie Ono, Kota Ishizawa, Tadashi Ishii

**Affiliations:** ^1^Shimizu Clinic, Sendai, Japan; ^2^Department of Kampo Medicine, Tohoku University Hospital, Sendai, Japan; ^3^Department of Education and Support for Regional Medicine, Tohoku University Hospital, Sendai, Japan; ^4^Department of Kampo and Integrative Medicine, Tohoku University Graduate School of Medicine, Sendai, Japan

**Keywords:** pancreatic cancer, Kampo medicine, integrative therapy, quality of life, prolong survival

## Abstract

**Aims:** The present report aims to investigate the use of Kampo medicine for advanced pancreatic cancer patients in order to prolong survival.

**Methods:** We retrospectively reviewed medical records of patients with pancreatic cancer who presented to our Shimizu Clinic from 2000 to 2020. Patients who survived at least twice as long as the initial prognostic estimate were selected and their treatment was reviewed. The Kampo formula and crude drugs were selected according to the Kampo diagnosis and treatment strategy, which included qi and blood supplementation; qi, blood and water smoothing; and inflammation (termed “heat”) and cancer suppression.

**Results:** Ten patients aged 45–80 years (six males and four females) with stage IV advanced cancer were selected. All patients received hozai, which is a tonic formula, of juzentaihoto (JTT) or hochuekkito (HET) decoction. Anti-cancer crude drugs were included in the decoctions of nine patients. At the first visit, the estimated life expectancy for all patients was no more than 1 year; however, treatment with Western and Kampo medicine led to a relatively long survival period of over 2 years. Three patients were still living at the time of this writing, more than 2, 6, and 14 years after treatment initiation.

**Conclusion:** Our results suggest that Kampo medicine may be useful for disease control and supportive care for patients with advanced pancreatic cancer.

## Introduction

Surgery, radiotherapy, and chemotherapy along with anticancer drugs are the standard Western treatments for pancreatic cancer. However, the therapeutic effects of these treatments are poor in cases of advanced disease, with a 5-year survival rate of 1.3% among patients with advanced pancreatic cancer (1). For patients with advanced cancer who have not responded to Western medicine, an integrated treatment approach using Kampo medicine may be a useful alternative. Kampo medicine is beneficial for the treatment of cancer-related numbness, constipation, anorexia, muscle cramps, and fatigue (2). Given this finding, the Japanese Society for Palliative Medicine has recommended the use of Kampo medicine and crude drugs in combination with Western medicine (3). Improvement of symptoms during cancer treatment may extend the patients' tolerance for longer treatment periods (4, 5).

We previously presented a case of a patient with advanced pancreatic cancer who survived for 7 years after diagnosis without a significant decrease in quality of life (QOL) (6). This initial case demonstrated that Kampo medicine may be useful for disease control and supportive care for patients with advanced pancreatic cancer. To study this idea further, we retrospectively reviewed cases of advanced pancreatic cancer seen in our clinic to summarise and evaluate the efficacy of Kampo treatment for this patient group.

## Methods

Medical records of 48 patients with advanced pancreatic cancer who were treated with Kampo medicine in our Shimizu Clinic from the year 2000 to the year 2020 were included in this retrospective review. Ultimately, 10 patients who lived at least twice as long as the estimated prognosis, determined at the time of the first visit, were enrolled for the study. The patients' age, sex, clinical stage of disease at the first visit, life expectancy at the first visit, Western medicine treatment, Kampo medicine treatment, and survival duration were collected from their medical records. The Kampo formula and crude drugs were selected according to the Kampo diagnosis and treatment strategy. This strategy entailed qi and blood supplementation; qi, blood and water smoothing; and inflammation (heat) and cancer suppression. The Kampo formulas of hozai and kuoketsuzai and anti-cancer drugs used for the patients are listed in Table 1. The concentration indicator and composition of each crude drug are regulated by Japanese Pharmacopoeia of Japan version 17th.

**Table 1:** Kampo formula, its constituents, and mechanisms according to prior studies.

**Kampo formula**	**Crude drug**	**Additional mechanisms for cancer and immune system according to prior studies**
Juzentaihoto (JTT)	Astragalus root, cinnamon bark, rehmannia root, peony root, cnidium rhizome, atractylodes lancea rhizome, angelica root, ginseng, poria sclerotium, glycyrrhiza	Prevention of malignant progression and tumour cell metastasis (7). Upregulation of T cell activities by decreasing Foxp3 (+) Treg populations (8). Enhancement of fluorouracil-induced myelosuppression (9).
Hochuekkito (HET)	Astragalus root, atractylodes lancea rhizome, ginseng, angelica root, bupleurum root, jujube, citrus unshiu peel, glycyrrhiza, cimicifuga rhizome, ginger	Enhancement of concomitant immunity against tumour development (10). Restoration of antitumor T cell responses by normalisation of serum corticosterone, interleukin (IL)-12, and costimulatory molecule expression (11). Maintenance of NK cell activity and suppression of stress mediators (12). Inhibition of proinflammatory cytokine production, particularly IL-6 (13). Enhancement of cisplatin-induced apoptosis (14). Inhibition of cytokine-mediated apoptosis or necrosis, resulting in a reduction of the gastrointestinal side-effects of cancer chemotherapy (15). B cell replenishment after radiotherapy (16).
Keppuchikuoto	Angelica root, peony root, cnidium rhizome, rehmannia root, bupleurum root, glycyrrhiza, peach kernel, platycodon root, safflower, achyranthes root, immature orange	Stimulation of IL-2 and tumor necrosis factor (TNF)-α secretion and enhancement of their immune function, resulting in tumour growth suppression (17).
	*Hedyotis diffusa*	Anticancer, antitoxic, and diuretic effects (18).
	*Scutellaria barbata*	Inhibition of growth several human cancers, including lung cancer, digestive system cancers, hepatoma, breast cancer, and chorioepithelioma (19).
	*Lobelia chinensis*	Inhibition of inducible nitric oxide synthase, cyclooxygenase-2, TNF-α, and IL-6 from the NF-κB pathway (20).

### Ethical Considerations

This case series was approved by the Institutional Review Board of the Tohoku University Graduate School of Medicine (Institutional Review Board No. 18910).

## Results

Ten patients between the ages of 45 and 80 years (six males and four females) with stage IV advanced cancer were selected (Table 2). All Kampo formulas were prescribed as decoctions. All patients received hozai, which is a tonic formula of juzentaihoto (JTT) or hochuekkito (HET). Anti-cancer crude drugs such as Hedyotis diffusa, Scutellaria barbata, and/or Lobelia chinensis were administered to patients 1 through 10, with the exception of patient 9. Patients 1, 2, and 8 received additional Kampo medicine for the relief of chemotherapy-related symptoms. At the first visit, the life expectancy of all patients was limited to no more than 1 year; however, treatment with Western medicine and Kampo medicine led to a relatively long survival period of over 2 years. Three patients undergoing Kampo treatment were still living at the time of this writing, more than 2, 6, and 14 years after treatment initiation.

**Table 2:** Patients with advanced pancreatic cancer who were treated with Kampo medicine and lived at least twice as long as the prognosis determined at the time of initial visit.

**Case number**	**Age (years)**	**Sex**	**Disease**	**Onset**	**Diagnosis method**	**Day of surgery**	**Surgery**	**Anti-cancer drug/radiation**	**First visit at clinic**	**Performance status at first visit**	**Stage at the first visit**	**Life expectancy at the first visit**	**Western medicine treatment**	**Kampo medicine treatment**	**Tumour marker trend**	**Survival**	**Current status**
1	45	M	Pancreatic cancer adenocarcinoma invasive ductal carcinoma, tub2	01/05/2003	Operation	11/06/2003	Distal pancreatectomy	1. Gemcitabine hydrochloride 2. Tegafur, gimeracil, oteracil potassium	25/08/2003	1	IVa	1 year	Surgery for primary tumour and lung metastases; chemotherapy; radiotherapy for brain metastases	JTT^†^ and Keppuchikuoto with *Hedyotis diffusa, Scutellaria barbata, Lobelia chinensis*, and Fructus Polygoni Orientalis for suppression of cancer and support of physical strength; Senpukuka-taishasekito, Goreisan, Corydails Tuber for symptoms of nausea, vomiting, headache, and vertigo.	Gradual increase	7 years	Death due to primary disease
2	74	F	Pancreatic cancer	N/A	CT	N/A	N/A	Gemcitabine hydrochloride	09/05/2006	3	IVb	3 months	Chemotherapy	JTT with *H. diffusa* for suppression of cancer and support of physical strength; Senpukuka-taishasekito for symptoms of nausea and vomiting.	Gradual decrease	3 years	Death
3	67	M	Pancreatic cancer, Gastric cancer, Thyroid cancer	N/A	Operation	26/11/2014	Distal pancreatectomy	Radiation	31/01/2015	2	IVb	1 year	Surgery for primary tumour and liver metastases	HET^‡^ with *H. diffusa* and Fructus Polygoni Orientalis for suppression of cancer and support of physical strength.	Gradual decrease	>6 years	Still alive
4	72	M	Pancreatic cancer	17/03/2013	Operation	8/4/2013, 9/8/2013	Distal pancreatectomy, Pancreatectomy	Tegafur, gimeracil, oteracil potassium	04/07/2013	2	IVa	1 year	Surgery for primary tumour and bile duct metastases	HET with *H. diffusa* and Fructus Polygoni Orientalis for suppression of cancer and support of physical strength; Inchinkoto for its cholagogic effect.	Gradual decrease	3 years	Death due to interstitial pneumonia
5	73	M	Pancreatic cancer, Lung cancer, Tongue cancer	01/09/1991	Operation	01/10/2006	Pancreatoduodenectomy	Gemcitabine hydrochloride	12/10/2006	1	IVb	1 year	Surgery for primary tumour; chemotherapy	HET with *H. diffusa* for suppression of cancer and support of physical strength; Inchinkoto for it cholagogic effect.	Gradual decrease	>14 years	Death due to pneumonia
6	61	M	Pancreatic cancer	01/12/2008	CT	N/A	N/A	Gemcitabine hydrochloride	20/07/2017	2	IVb	6 months	Chemotherapy	HET with *H. diffusa* for suppression of cancer and support of physical strength.	Gradual increase	6 years	Death
7	68	F	Pancreatic cancer	04/04/2015	CT	15/10/2015	Pancreatic head resection	Gemcitabine hydrochloride	20/01/2019	2	IVb	1 year	Surgery for primary tumour; chemotherapy	HET with *H. diffusa* for suppression of cancer and support of physical strength.	Gradual increase	>2 years	Still alive
8	80	F	Pancreatic cancer	16/05/2017	CT	01/06/2017	Primary inoperable, bile duct stenting	Gemcitabine hydrochloride	13/06/2017	2	IVb	6 months	Surgery for primary tumour; chemotherapy	JTT with *S. barbata* and Fructus Polygoni Orientalis for suppression of cancer and support of physical strength. Senpukuka-taishasekito for symptoms of nausea and vomiting.	Gradual decrease	3 years	Death
9	62	M	Pancreatic cancer	01/02/2017	MRCP	N/A	Inoperable	1. Paclitaxel 2. Gemcitabine hydrochloride	27/04/2017	1	IVb	6 months	Chemotherapy	HET for support of physical strength.	Gradual increase	3 years	Death
10	78	F	Pancreatic cancer	25/05/2016	MRI	N/A	Inoperable	N/A	18/06/2016	2	IVb	1 year	Palliative care only	HET with *H. diffusa* and *S. barbata* for suppression of cancer and support of physical strength.	Gradual increase	3 years	Death

*N/A, not assigned; CT, Computed Tomography; MRCP, Magnetic Resonance cholangiopancreatography; MRI, Magnetic Resonance Imaging*.

† *JTT, Juzentaihoto*;

‡ *HET, Hocuekkito*.

## Discussion

The cases presented herein indicate that Kampo medicine can be provided for advanced pancreatic cancer treatment as an integrative cancer therapy. Additionally, these cases suggest that Kampo medicine may slow cancer progression and improve the survival rate. The treatment strategy for advanced cancer patients includes supporting vital energy and nutrition, harmonising the immune system and sympathetic condition, suppressing inflammation, and promoting microcirculation and interstitial fluid. These concepts are expressed as balancing qi, blood, and fluid or cold and heat within Kampo theory. Anti-cancer drugs attack cancer cells but also cause body damage, which reduces body recovery and innate immunity. On the other hand, Kampo medicines act on biological reactions and they have a supplementary effect on recovery and reduced immune system. This characteristic is important for striking a balance between offence against cancer and defence for the whole body.

Hozai, such as JTT or HET, are used to support vital energy and nutrition and to harmonise the immune system and sympathetic conditions. The indications for JTT include declined constitution after recovery, fatigue and malaise, anorexia, and anaemia. Additional effects of JTT on suppression of cancer growth, including prevention of malignant progression and tumour cell metastasis (7), upregulation of T cell activity (8), and improving fluorouracil-induced myelosuppression (9) have been reported in several experimental studies. HET has been reported to reduce cancer-related fatigue and improve QOL for cancer patients (21, 22). Additional reported effects of HET on cancer include concomitant enhancement of immunity against tumour development (10); restoration of antitumor T cell responses, and costimulatory molecule expression (11); maintenance of NK cell activity and inhibition of stress mediators (12); inhibition of proinflammatory cytokine production (13); enhancement of cisplatin-induced apoptosis (14); and inhibition of cytokine-mediated apoptosis or necrosis, leading to a reduction of the gastrointestinal side effects of cancer chemotherapy (15); and replenishing B cells after radiotherapy (16). These reports support the possibility of suppressing tumour growth and affecting immunomodulation to reduce inflammation in addition to the original supportive effects for fatigue and malaise. HET also can promote negative conversion of vancomycin-resistant Enterococci or prevent the colonisation of methicillin-resistant Staphylococcus aureus in humans (23, 24). These studies suggested that HET influenced on innate immunity and nutrition status.

Kuoketsuzai, which is a blood stasis-resolving formula such as Keppuchikuoto, has been used for relief of pain caused by blood stasis (25). It stimulates interleukin (IL)-2 and tumor necrosis factor (TNF)-α secretion and improves immune function, resulting in tumour growth suppression (17). For pain control, some crude drugs were added to the formula. Corydalis tuber, a crude drug that contains isoquinoline alkaloids, is used for intractable pain and can be used to manage pain associated with bone metastases.

Some herbal medicines may be added to Kampo treatment due to their anti-cancer effects. *H. diffusa* is used as an anticancer, antitoxic, and diuretic agent to treat cancers (18). Extracts of *S. barbata* have inhibitory effects on the growth of several cancers in humans, including lung cancer, gastrointestinal cancers, hepatoma, breast cancer, and chorioepithelioma (19). *L. chinensis* has anti-inflammatory properties that are attributable to inhibition of inducible nitric oxide synthase, cyclooxygenase-2, TNF-α, and IL-6 *via* the NF-κB pathway (20). The combination of hozai with anti-cancer crude drugs may support patient condition and inhibit cancer growth, resulting QOL and relatively prolonged survival rate.

In 2019, the 11th revision of the International Statistical Classification of Diseases and Related Health Problems published by the World Health Organisation included a traditional medicine module (26). Following this global trend, recently, most of the clinical practice guidelines in Japan recommended Kampo medicines for symptoms and diseases (27–29). Our previous report suggested that integrative medicine combined with Kampo medicine and western medicine can be applied for several intractable symptoms and diseases (6, 30–34). Therefore, Kampo treatment may be a helpful tool during advanced pancreatic cancer treatment.

Nowadays, the term “integrative oncology” is used for multidisciplinary cancer treatment. It includes a combination of complementary medicine in conjunction with conventional cancer treatments (35). Complementary medicine and traditional medicine have been incorporated to the contents of the Basic Medical Education: Japanese Specifications WFME (World Federation for Medical Education) Global Standards for Quality Improvement (36). It showed to have an opportunity to contact complementary medicine and traditional medicine in Japan. Furthermore, Model Core Curriculum for Medical Education revised at 2017 included the objectives of outlining the characteristics of Kampo medicine, the indications and pharmacological effects of major Kampo medicines (37). Considering these concepts and educational process, it is important to understand the characteristics of Kampo medicines and use them effectively along with conventional cancer treatments.

This research has some limitations. The study design is a case series; we did not include control or comparison groups. In the retrospective analysis, we did not have complete data of patients' entire clinical course. Thus, we could not compare the factors relating to prognosis between delayed prognosis and poor prognosis. Further, study will be needed to clarify the factors relating to prognosis including Kampo treatment.

In conclusion, Kampo medicine may be useful for disease control and supportive care for patients with advanced pancreatic cancer and may result in a relatively prolonged survival rate.

## Data Availability Statement

The raw data supporting the conclusions of this article will be made available by the authors, without undue reservation.

## Ethics Statement

This case series was approved by the Institutional Review Board of the Tohoku University Graduate School of Medicine (Institutional Review Board No. 18910). Written informed consent for participation was not required for this study in accordance with the national legislation and the institutional requirements.

## Author Contributions

MS treated patients. MS and ST wrote manuscript. AK, RO, and RA selected patients from medical records. KI and TI revised manuscript. All authors contributed to the article and approved the submitted version.

## Conflict of Interest

ST, AK, and TI belong to the Department of Kampo and Integrative Medicine at the Tohoku University School of Medicine. The department received a grant from Tsumura, a Japanese manufacturer of Kampo medicine; however, this grant was used as per Tohoku University rules. Potential conflicts of interest were addressed by the Tohoku University Benefit Reciprocity Committee and managed appropriately. The remaining authors declare that the research was conducted in the absence of any commercial or financial relationships that could be construed as a potential conflict of interest.

## Publisher's Note

All claims expressed in this article are solely those of the authors and do not necessarily represent those of their affiliated organizations, or those of the publisher, the editors and the reviewers. Any product that may be evaluated in this article, or claim that may be made by its manufacturer, is not guaranteed or endorsed by the publisher.
